# Prevalence of asthma and level of fractional exhaled nitrogen oxide among Malaysian school children

**DOI:** 10.1186/1471-2458-14-S1-O27

**Published:** 2014-01-29

**Authors:** Aminnuddin Ma'pol, Jamal Hisham Hashim, Dan Norbäck, Gunilla Weislander, Zailina Hashim, Zaleha Md Isa

**Affiliations:** 1 Jabatan Kesihatan Masyarakat, Pusat Perubatan Universiti Kebangsaan Malaysia, Jalan Yaacob Latif, Bandar Tun Razak, 56000 Cheras, Kuala Lumpur, Malaysia; 2 United Nations University International Institute for Global Health (UNU-IIGH), 56000 Cheras, Kuala Lumpur, Malaysia; 3 Uppsala University, Uppsala, Sweden; 4 Putra University, Selangor, Malaysia

## Background

School classrooms remain one of the commonest places where outdoor and indoor air pollution can accumulate. The aim of the present study was to compare the level of fractional exhaled nitrogen oxide (FeNO) across sex, asthma and allergy status among 14 year-old Malaysian school children.

## Materials and methods

The use of FeNO measurements in clinical practice has been increasingly accepted based on a number of theoretical and practical factors. It is not only a user friendly, portable and non-invasive assessment tool, but is also able to detect inflammatory airway changes in children exposed to atmospheric pollutions. The study respondents were randomly selected from secondary school students in Terengganu, Malaysia. A questionnaire with previously tested questions was used to obtain information on the respondents’ living conditions, school environment, asthmatic symptoms and allergies. FeNo measurement was conducted using a NIOX-MINO (50 ml/min flow) instrument.

## Results

From 481 students, only 361 students were given consent by their parents for medical and physical examinations. A majority (99%) of the respondents were Malays, 63% were females and 68% stayed in single houses made from bricks. The major asthmatic-related complaint was breathing difficulty induced by severe physical activity (58%). The prevalence of doctor diagnosed asthma was 7%, of which 38% of students with known asthma were on medication. Although, there was no significant difference in the FeNO levels between asthmatic and non-asthmatic students (28±3.2ppm versus 21±1.4ppm), but FeNO levels were higher among boys (24±1.6ppm versus 20±1.1ppm), and students in the urban schools (24±1.5ppm versus 19±1.1ppm).

## Conclusions

The effect of hot and humid climate on the level of exhaled FeNO in Malaysia is a relatively new issue.

